# Advances and Challenges in Cancer Stem Cells for Onco-Therapeutics

**DOI:** 10.1155/2023/8722803

**Published:** 2023-12-06

**Authors:** Sulaiman Mohammed Alnasser

**Affiliations:** Department of Pharmacology and Toxicology, Unaizah College of Pharmacy, Qassim University, Qassim 51452, Saudi Arabia

## Abstract

Six decades have passed since the foundational recognition of the primary properties of the stem cells. Research on stem cells has since remained at the forefront of efforts to combat a spectrum of diseases, most notably cancer. Despite remarkable progress in medical science, a definitive cure for cancer has remained elusive, spurring the pursuit of diverse therapeutic strategies, among which stem cell therapy is a particularly promising avenue. Moreover, the utilization of cancer stem cells as a therapeutic source holds immense potential for addressing intractable diseases. The strategy of targeting cancer stem cells is beset with challenges, including immune rejection and disease relapse. Additionally, the capacity to inadvertently generate cancer stem cells upon transplantation underscores the critical need to eliminate these cells to ensure the efficacy of cell-based therapies. This paper underscores the pivotal role of cancer stem cells in onco-therapeutics and their potential to aid in early cancer diagnosis. With the proliferation of tissue banks and their collection of malignant tissue types, a renewable source of medications to combat cancer is on the horizon. While cancer stem cell-based therapy presents sophisticated and significant challenges, it offers unprecedented opportunities to extend human life. Continued technological advancements in stem cell therapy promise to provide new insights and refine approaches for cancer treatment, ushering in a new era of hope and innovation in the fight against this formidable disease.

## 1. Introduction

Cancer stem cells (CSCs), also known as tumor-initiating cells, represent a small subset of cells within a tumor with distinct properties. Unlike the bulk of cancer cells, CSCs possess stem cell-like characteristics, including the ability to self-renew and differentiate into various cell types found within the tumor [1]. Tumors lacking stem cells exhibit varying degrees of cell population differentiation but still display a high-proliferation rate [2]. CSCs are believed to be at the root of tumor initiation, progression, and therapy resistance. Their capacity to evade current cancer treatments makes them a focus of intense research as the potential to develop into cancer has raised concerns regarding their therapeutic applications [3]. Various factors are considered to be crucial in the “transformation” of stem cells, resulting in CSCs, and are believed to include a combination of stochastic and hierarchical factors [4, 5]. Owing to their ability to self-renew and differentiate into various cell types, stem cells tend to accumulate mutations and epigenetic influences over time. They can spread and become more dangerous regardless of their location because they have been shaped by the natural selection to do well in harsh environments [4].

The intricate molecular mechanisms governing the regulation of CSCs self-renewal and differentiation in diverse cancer types entail a comprehensive network of transcription factors and signaling pathways. Transcription factors, including OCT4, Sox2, Nanog, and KLF4, serve as orchestrators of this regulatory paradigm by modulating gene expression and sustaining the pluripotent state of CSCs [6]. Simultaneously, signaling pathways, such as Wnt, Notch, Hedgehog, JAK-STAT, PI3K/AKT/mTOR, TGF/SMAD, and PPAR, play indispensable roles in the intricate orchestration of CSC behavior [7]. The Wnt pathway, for example, reinforces stemness by stabilizing *β*-catenin, thus augmenting self-renewal capacity [8]. In parallel, Notch and Hedgehog pathways maintain stemness and curtail differentiation [9]. The JAK–STAT pathway governs the delicate equilibrium between survival and proliferation of CSCs, while the PI3K/AKT/mTOR pathway wields influence over their metabolic processes and self-renewal potential [10]. The context-dependent duality of TGF/SMAD signaling manifests either as a promoter or an inhibitor of CSC self-renewal [11]. In addition, the activation of peroxisome proliferator-activated receptors (PPARs) steers CSCs toward differentiation [12]. These intricate molecular components collectively form a dynamic and complex regulatory network dictating the multifaceted development and functionalities of CSCs within the context of cancer. The equilibrium between self-renewal and differentiation, meticulously regulated by these mechanisms, is pivotal for tumor growth and progression. A profound comprehension of this intricate molecular interplay is imperative for the development of targeted therapeutic modalities aimed at selectively eradicating CSCs, thereby mitigating the risk of cancer relapse.

Cancers originating from malignant stem cells are particularly concerning due to their resistance to the conventional anticancer medications, as even a single surviving CSC can lead to tumor recurrence [13]. Furthermore, it has been postulated that these tumor cells may be protected by altered microenvironmental habitats compared to the normal stem cell niche [14]. Targeting CSCs has the potential to revolutionize cancer treatment and significantly enhance patient outcomes. By eradicating the cells responsible for therapy resistance, recurrence, and metastasis, the chances of achieving long-term remission and improving overall survival rates are greatly enhanced. The development of therapies that specifically target CSCs has the potential to transform cancer treatment and provide new hope for patients facing this formidable disease. In order to establish novel treatment strategies that stop the growth of cancer, Zarrintaj et al. [15] conducted a review to identify the biological distinctions between healthy and CSCs, as well as to comprehend the mechanisms that govern these distinctive mechanisms. Experimentally, the frequency of CSCs is typically considered to be modest—less than 1% in unfractionated cancer cell populations [16].

Despite reported advancements, researchers must exercise prudence when pursuing a treatment strategy. The biology of stem cells is still in its infancy, and fresh information refuting or confirming existing understanding emerges daily. As research progresses from the laboratory to the clinic, it is crucial to give significant consideration to the social, ethical, and political implications surrounding it. While it may not be possible to address all the concerns raised, it is vital to engage in ongoing discussions and work in parallel. Researchers can only achieve a more controlled balance between self-renewal and cell differentiation, stimulating tissue regeneration, by understanding the molecular processes that regulate cell division. Furthermore, clearer recommendations for the optimal use of cell (and gene) therapy are necessary to enhance the quality of life for individuals [17]. It is anticipated that novel stem cell therapies will replace current, more expensive, and often ineffective treatments. Additionally, stem cells are considered crucial experimental models for studying cell differentiation, embryonic development, cancer mechanisms, and other areas. This growing understanding of fundamental biology may lead to improvements in the existing treatment methods for human and animal disorders in the near future [18–20]. Hence, this review underscores the significance of CSCs in disease therapy.

## 2. Difference between Cancer and CSCs

In the 1950s and 1960s, Till Thompson and McCulloch [21] and Sornberger [22] of Toronto performed pioneering work in the field of CSCs. John Dick's research team discovered that acute myeloid leukemia (AML) has a leukemia stem cell fraction with the same surface markers as normal hematopoietic stem cells. Only these CSCs could induce AML in immunocompromised mice [21, 22]. Rossi et al. [23] conducted a detailed analysis of the distinctions between cancer epithelium and CSCs (Table 1). CSCs were first discovered in leukemia and breast cancer tissues. They have the ability for allografting, resistance to therapy and metastasis [35, 36].

In breast cancer, transplanting a few hundred CSCs can produce a tumor, whereas transplanting a few hundred thousand “normal” cancer cells does not [37]. Biopsies of human carcinomas from brain tumors [38], colon carcinomas [39], and head and neck cancer [40] yielded similar findings. In each of these experiments, the transplanted CSC fraction was able to grow tumors in immunocompromised animals with the original histology. CSCs have also been detected in lung carcinoma [41], pancreatic carcinoma [42], and malignant melanoma [43]. Due to their resistance to apoptosis, neither chemotherapy nor radiation can effectively destroy the majority of CSCs [44]. Chemotherapy and radiation can reduce the size of a tumor, but only the aggressive cells survive [37]. This is why it is common for a particularly aggressive recurrence to occur following remission. Therefore, to effectively treat cancer, the therapy would have to target tumor stem cells. Further investigations have demonstrated the presence of CSCs in CML patients treated with imatinib [45, 46]. Stem cells can enter a latent state that is resistant to cytostatics, utilize detoxifying transport channels, and activate antiapoptotic signaling pathways to protect themselves from cell death.

## 3. The Persistence of Cancers and CSCs

Tumorigenesis involves the accumulation of numerous mutations, which, influenced by natural selection, result in the proliferation of more aggressive cell subpopulations and contribute to tumor progression [25]. Although this notion is well-established, the experimental identification of cells capable of generating tumors has just recently begun. When implanted into immunocompromised mice, a small number of cancer mass cells can multiply and form new tumors, as observed in breast cancer, prostate cancer, and leukemia [2, 47]. These cells are referred to as CSCs, and they share several traits with normal stem cells. Both cell types are capable of self-renewal, sustaining the stem cell population indefinitely, and producing cells capable of differentiating into at least one lineage. As differentiation progresses, the proliferative rate decreases, rendering terminally differentiated cells unable to multiply and prone to activating the apoptotic program after a specific period. Consequently, the majority of the cells in the tumor mass are not tumorigenic. Thus, tumors consist of varied cells at different stages of growth, making them different from one another [48].

Given that stem cells rely on a specific microenvironment to maintain their capacity for self-renewal and that peritumoral tissues influence the maintenance of the tumor state, detailed studies on the influence of microenvironments on the maintenance of CSCs are essential for comprehending the biology of cancers. Two fundamental stem cell characteristics that influence tumor growth are a low-proliferative rate and high expression of multidrug resistance proteins. These traits indicate that traditional chemotherapy, which mainly targets proliferating cells, would be ineffective in eliminating these cells. Therefore, the identification of CSCs with distinct molecular pathways and a better understanding of the tumor microenvironment could lead to the development of targeted therapies that are less aggressive and more effective in eliminating the cells responsible for tumor growth [47, 49]. Such advancements would decrease the likelihood of cancer relapse.

## 4. Diapause and Hibernation Mechanism in CSCs

When cancer cells are exposed to chemotherapy, they undergo hibernation or senescence [50]. Researchers have tested ATR protein inhibitors on AML organoids and mouse models, successfully preventing cancer cells from entering hibernation when administered before chemotherapy. This approach shows promise in improving the effectiveness of chemotherapy for breast cancer, prostate cancer, GI cancer, and other types [51]. Furthermore, studies have revealed that certain cancer cell subtypes capable of entering hibernation can contribute to the cancer recurrence. This highlights the importance of targeting these hibernating cancer cells to prevent disease relapse and improve treatment outcomes. Cancer cell subtypes that comprehend “hibernation” can induce cancer recurrence [52]. Although further research is needed to unravel the intricate mechanisms of diapause and its implications in cancer biology, the exploration of this biological phenomenon opens new avenues for improving the cancer therapies. By deciphering the molecular mechanisms that regulate diapause and developing strategies to selectively target hibernating cancer cells, researchers strive to overcome treatment resistance, prevent tumor recurrence, and ultimately improve patient outcomes in the fight against cancer [53].

## 5. Filling Knowledge Gaps on CSCs through Research

Recently, the understanding that stem cells exist in all tissues has been extended to cancer. The roles of stem cells in normal tissues have now been linked to oncogenesis, highlighting the significance of targeting and altering CSCs as a crucial treatment strategy [54]. In recent years, the identification of stem-like cells in tumors has become a major aspect of cancer cell biology research. As with any new field, there is currently no consensus on how to identify tumor stem cells in a population of diverse tumor cells. Various methods such as marker presence detected through flow cytometry, immunodetection, or RT-PCR, as well as functional characteristics like sphere formation or growth in animals, are being utilized [55, 56]. This concept of CSC attempts to suggest that not all forms of growth within tumors are responsible for sustaining tumor growth or initiating new growth. Rather, there is a distinct fraction of malignant cells known as CSCs that possess stem-like characteristics, including self-renewal and differentiation abilities. These malignant cells play a crucial role in tumor progression and metastasis. In the case of AML, evidence supporting the existence of leukemic stem cells (LSCs), a type of CSC, was initially discovered in 1997 [57]. Since then, most investigations have focused on three key aspects: (a) the ability of CSCs to engraft and initiate tumor growth; (b) their capacity for serial transplant growth and the ability to regenerate tumors following transplantation into immunocompromised mice; and (c) their heterogeneity and ability to give rise to non-CSC offspring. Many diseases exhibit phenotypic and functional similarities between malignant tissues and CSCs [57, 58]. The expression patterns of markers in AML-LSCs resemble those observed in normal hematopoietic stem cells or the parent tissues from which malignancies arise. Similarly, colorectal CSC populations exhibit gene expression profiles similar to those of normal adult intestinal tissue stem cells, and this similarity has been successfully replicated in an immunocompromised mice [59]. The presence of a CSC-like signature in breast and colorectal cancers indicates the aggressive nature of these diseases. Recent studies have also explored the integration of gene expression and epigenetic features in various human malignancies. CD47, a protein present in both cancerous and noncancerous tissues, has been identified as a potential target for CSCs [60]. High expression of CD47 in AML–LSCs has been associated with shorter overall survival in AML patients. In allograft transplant experiments, the use of anti-CD47 antibodies reduced the growth of human AML in immune-deficient mice, demonstrating the significant role of CD47 in LSC-driven proliferation [61]. Furthermore, treatment with anti-CD47 antibodies in mice with human AML led to a significant reduction in circulating AML–LSCs and a substantial decrease in LSCs within the bone marrow. Subsequent transplantation of cells from anti-CD47-treated mice did not result in leukemia engraftment, indicating successful eradication of AML–LSCs [62].

The mechanism that allows CSCs to evade the immune system and produce tumors in other organs, known as metastases, was uncovered in a study released by researchers at Princeton University in the United States. CSCs acquire new characteristics through genetic pathways that are typical of normal stem cells. This enables them to adapt and become more aggressive, ultimately playing a role in tumor instigation, metastasis, and treatment resistance [63]. Scientists at Cornell University developed nanoparticles that circulate in the bloodstream and selectively destroy cancer cells upon contact, specifically cancer cells that have spread from the primary tumor and formed metastases [64]. Furthermore, research has focused on the role of microRNAs in the differentiation of prostate CSCs. This investigation has allowed for the analysis of their sensitivity to conventional and natural medications, as well as the identification of pathways involved in differentiation. Additionally, it has provided insights into the potential for cancer metastasis and the identification of microRNAs that undergo changes during the differentiation process [65, 66].

## 6. CSC Models

CSC models are experimental systems that aim to mimic the behavior and characteristics of CSCs in a controlled laboratory environment. These models are essential for studying the biology of CSCs, investigating their role in tumor initiation, progression, and therapy resistance, as well as developing novel therapeutic strategies. These include:

1. Cell line-derived models: cancer cell lines derived from tumor samples or established cell lines can be used to generate CSC models. These models involve isolating and enriching cells with stem-like properties, such as self-renewal and differentiation potential. By studying these cell populations, researchers can gain insights into CSC biology, identify CSC-specific markers, and investigate their response to the various treatments [67, 68]. Cell line-derived models offer numerous advantages for scientific research. The ease of maintenance, coupled with their cost-effectiveness, makes them accessible to a wide range of research laboratories. Many cell lines exhibit rapid growth, facilitating high-throughput studies, and some are amenable to genetic manipulation, enabling the investigation of specific gene functions. Additionally, the use of cell lines avoids ethical concerns associated with animal or human research, making them a practical choice for various experimental settings [69]. Despite their advantages, cell line-derived models come with limitations. They do not fully capture the complex *in vivo* microenvironment and interactions present within a whole organism, which can lead to discrepancies between experimental outcomes and physiological reality. Many cell lines are derived from a limited range of tissue types, limiting their representativeness. Moreover, cell lines can undergo phenotypic and genetic drift, potentially deviating from the original tissue or tumor characteristics. They lack interactions with the extracellular matrix and lack physiological relevance, residing in artificial conditions. Finally, their responses to drugs may not accurately reflect in vivo responses, which can lead to misleading conclusions in drug development and testing [70].
2. Patient-derived xenografts (PDX): PDX models involve implanting patient-derived tumor tissues or CSC populations into immunodeficient mice [71]. These models better recapitulate the tumor microenvironment and allow the study of tumor growth, metastasis, and therapeutic response in vivo. PDX models retain the heterogeneity and genetic characteristics of the original tumor, making them valuable tools for preclinical drug testing and personalized medicine approaches [72–74]. PDX models offer significant advantages in mimicking human tumors and assessing drug responses. However, they require substantial resources, lack a human immune system, and may not be feasible for all tumor types [75]. Researchers should carefully consider these pros and cons when choosing PDX models for their specific research objectives.
3. 3D culture systems: 3D culture systems, such as tumor spheroids or organoids, aim to mimic the three-dimensional architecture and cellular interactions within tumors. In 1907, Wilson [76] conducted a groundbreaking experiment demonstrating the remarkable regenerative potential of sponge cells. This was the start of organoid development technology and since then, stem cell researchers have made significant progress in generating organoids from stem cells to study various types of cancer, including breast [77], lung [78], colon [79], and pancreatic ductal adenocarcinoma [80]. Translational models of stem cells and induced pluripotent stem cell organoids for gastric cancer are already in place to study gastric cancer [81]. These models provide a more physiologically relevant environment for studying CSC behavior, including self-renewal, differentiation, and response to therapies. Organoids derived from precancerous lesions serve as valuable models for understanding tumor development and analyzing tumor-related changes while tumor organoids exhibit characteristics similar to the original tissue and retain the heterogeneity observed in individual cancers [82]. This feature makes them promising tools for precision medicine and translational research, offering a powerful platform to study cancer biology and explore personalized treatment options. 3D culture systems can be derived from patient samples or established cancer cell lines [83–85]. For instance, patient-derived organoids in high-grade serous ovarian carcinoma have been successfully used to study different mutational processes driving chromosomal instability, such as homologous recombination deficiency, chromothripsis, tandem-duplicator phenotype, and whole genome duplication [86]. They can be used for testing compound sensitivity, and guide the development of precision therapeutics. In colorectal carcinoma organoids, differential responses to various agents, including oxaliplatin and palbociclib have been studied [87]. Combined with other techniques like RNA-seq and mass-spectrometry, colorectal carcinoma organoids have the potential to predict treatment response and aid personalized cancer therapy development.

However, despite their potential, there are several limitations associated with organoid research in the context of cancer. The success rates of generating organoid models vary greatly, and the conditions for organoid culture require optimization to enhance reproducibility [88]. The absence of certain cell types, such as stromal cells, in organoids hinders their ability to accurately predict clinical outcomes and evaluate the efficacy of immunomodulatory treatments [82]. Moreover, current organoid models lack vascularization and the ability to model interactions between different tissues and organs, limiting their ability to fully recapitulate the complexity of *in vivo* environments [89]. Standardization of protocols and cost-effective production methods are also essential for the broader adoption of organoid technology in the healthcare systems [88, 90]. Therefore, organoids have significant potential in cancer research but addressing these limitations and advancing organoid technology will be crucial to unlock their full potential as reliable preclinical and clinical models for drug screening and personalized medicine.

(4) Genetically engineered mouse models (GEMMs): GEMMs involve manipulating the genetic makeup of mice to develop specific cancer types or target specific genes associated with CSCs. These models allow researchers to study the role of specific genetic alterations in CSC formation, tumor initiation, and progression. GEMMs can also be used to evaluate the efficacy of targeted therapies against CSCs in a more complex and dynamic system [91]. They offer precise genetic control and closely mimic human diseases, making them invaluable for many research applications [92]. However, they are resource-intensive, may not be suitable for all diseases, and raise ethical considerations.
(5) Organotypic models: organotypic models aim to recreate the complexity of the tumor microenvironment by combining multiple cell types and extracellular matrix components [93]. These models can include cocultures of CSCs with other cell types, such as stromal cells, brain cells [94], or immune cells, to investigate their interactions and their influence on CSC behavior. Organotypic models provide a platform to study CSC-mediated tumor-stroma interactions, immune evasion mechanisms, and potential therapeutic interventions [95]. Organotypic models offer a highly relevant platform for studying tissue-specific diseases and drug responses with the reduced ethical concerns. However, they can be resource-intensive, may lack long-term viability, and exhibit variability when using patient-derived samples [96].
(6) Organ-on-a-chip technology: organ-on-a-chip is a rapidly evolving field that involves the development of microscale devices that mimic the structure and function of human organs [97]. These devices typically consist of microfluidic channels lined with living cells that replicate the physiological environment of a specific organ or tissue. In the context of CSC research, organ-on-a-chip platforms can be used to recreate the microenvironment of tumors and study the behavior of CSCs as researchers can create a microfluidic chip that mimics the blood vessels and surrounding tissues of a specific organ affected by cancer [98]. By introducing CSCs into this system, researchers can observe how the cells interact with their environment, migrate, invade surrounding tissues, and respond to different treatment modalities [99]. Organ-on-a-chip technology can create more realistic and physiologically relevant cancer models compared to traditional 2D cell culture systems. It allows for the integration of multiple cell types, dynamic fluid flow, and the application of mechanical forces, providing a more comprehensive understanding of CSC behavior [100]. Overall, organ-on-a-chip technology offers highly detailed and physiologically relevant models for studying organs and tissues. However, it can be complex and costly, and achieving standardization can be challenging [101].
(7) Spheroids: spheroids are 3D-cell culture models that closely resemble the structure and behavior of tumors *in vivo*. They are typically formed by growing cancer cells in suspension or embedding them in an extracellular matrix, allowing the cells to aggregate and form compact, spherical structures. Spheroids can be derived from CSCs or bulk cancer cell populations. When using CSCs, spheroids can help to maintain the stemness and heterogeneity of the cell population, allowing researchers to study the characteristics and behavior of CSCs in a more physiologically relevant setting. Spheroids derived from bulk cancer cell populations can also provide insights into the interactions between CSCs and other tumor cells within a 3D microenvironment and investigate self-renewal, differentiation, invasion, metastasis, and drug response behavior [98]. Various types of spheroids have been established for different cancer types, such as glioma-derived spheroids or neurospheres, mammalian cancer spheroids (mammospheres), and colorectal cancer spheroids (colonspheres) [93, 102–104]. Neurosphere CSCs closely resemble the genotype, gene-expression profile, and biology of the parental tumors [93]. Lee et al. [102] have demonstrated the ability to generate different types of mature neural cells and exhibited multilineage differentiation when transplanted *in vivo*. Mammospheres have also been derived from metastatic cells and ductal carcinoma *in situ*, and they have been utilized to investigate intertumoral heterogeneity, signaling pathways, and the effects of chemical compounds on CSCs [93, 103]. Colonospheres have reproduced the histopathological features of the original tumor when transplanted into mice and have been extensively used to study CSC-related characteristics such as chemoresistance, metastatic capacity, and tumorigenicity at the single-cell level [93, 104].

Scientists have demonstrated that *ex vivo* drug sensitivity testing in 3D spheroidal cultures accurately replicates clinical responses to chemotherapy and immunotherapy, particularly in cisplatin-based chemotherapy and anti-PD-1 therapy in lung cancer [105]. This indicates the promising potential of using these culture models to predict patient outcomes, facilitating the selection of individualized therapies. By assessing the drug sensitivity of patient-derived tumor spheroids, researchers have obtained consistent correlations with the patient's actual clinical response to these treatments. Spheroids capture important aspects of tumor biology, including cell–cell interactions, nutrient and oxygen gradients, as well as resistance to therapies [106, 107]. Spheroids can be analyzed using techniques like microscopy, gene expression profiling, and drug screening assays to assess the effects of different treatments on CSCs.

Each CSC model has its advantages and limitations, and the choice of model depends on the specific research question and experimental design. By utilizing these models, researchers can gain insights into the biology and behavior of CSCs, identify novel therapeutic targets, and develop more effective treatment strategies to combat cancer.

## 7. Role of CSCs in Clinical Findings

**Figure 1:**
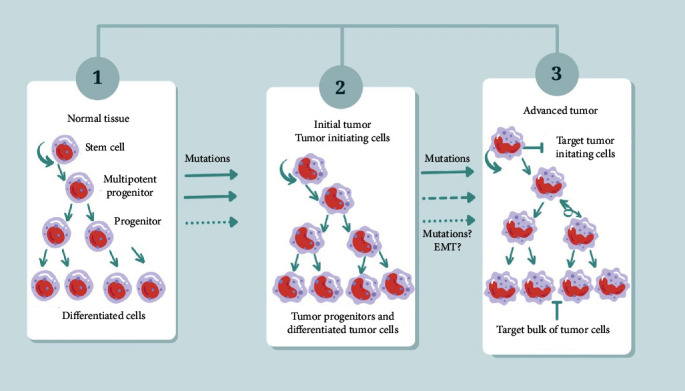
Tumor initiation cells known for their ability to initiate tumor formation due to mutations, serve as the “seeds” of malignancy. These cells allow cancer to take root and propagate. Tumor initiation cells are often associated with the process of epithelial–mesenchymal transition (EMT), a critical event in cancer progression. This figure is adapted from the study of Zhou et al. [108].

CSCs can serve as biomarkers for the early detection, diagnosis, and prognosis of various types of cancer. CSCs can also aid in drug radiation resistance and tumor initiation (Figure 1) [109].

Their presence and characteristics can provide valuable insights into tumor aggressiveness, treatment response, and likelihood of recurrence. Identifying and targeting CSC-specific biomarkers can aid in personalized treatment strategies and monitoring disease progression. SCs can be utilized for drug screening and testing novel therapeutic agents. By culturing CSCs *in vitro* or developing animal models with CSC populations, researchers can assess the efficacy of different drugs and identify potential candidates for the further clinical development. This approach enables the identification of drugs that specifically target CSCs, ultimately leading to more effective treatment options.

Researchers have discovered new biomarkers in CSCs that govern the survival and spread of cancer, and hope is rising that drug discovery to kill CSCs can follow suit (Table 2) [110–113]. Biomarkers can help clinicians detect that an abnormal process may be underway and can appear as an array of aberrant proteins, such as hormones, enzymes, or signaling molecules, and may vary from patient to patient.

Studying CSCs can provide insights into the mechanisms underlying tumor heterogeneity and clonal evolution, which can guide the development of more effective treatment strategies. By targeting CSCs, it may be possible to disrupt tumor growth, inhibit metastasis, and prevent the development of therapy-resistant cell populations. It is important to note that while the clinical uses of CSCs show significant potential, further research is needed to fully understand their biology, behavior, and therapeutic implications. Ongoing studies and clinical trials are focused on unraveling the complexities of CSCs and translating this knowledge into improved patient care and outcomes in the fight against cancer. Clinicians can make informed decisions through these methods regarding treatment options, expectations as well as monitoring.

## 8. Treatment and Side Effects Associated with Stem Cell Therapy

One of the promising approaches in cancer treatment is targeting CSCs specifically [114]. Conventional cancer therapies such as chemotherapy and radiation primarily target rapidly dividing cells, but they may not effectively eliminate CSCs, which are often more resistant to these treatments [115]. CSCs can survive the initial therapy and contribute to tumor recurrence and metastasis. It is crucial that the accurate separation and recognition of malignant tissues be programed and designed, which entails understanding mechanisms that regulate the expansion of cancer stem cell colonies and the development of drug resistance in order to design effective tailored therapies. The immunomodulation, immune evasion, and impact resistance brought about by CSCs significantly alter the ability of the natural immune system to work in harmony [116, 117]. In tumor progression, signaling via mTOR (mammalian target of rapamycin), SHH (sonic hedgehog), notch receptor, and Wnt/*β*-catenin can be harnessed for modulating tumor progression [118]. Additionally, CSC-based therapies include the development of drugs that selectively target CSCs or disrupt the supportive microenvironment that promotes their survival [25, 119]. These therapies aim to inhibit the self-renewal capabilities of CSCs, induce their differentiation into noncancerous cell types, or sensitize them to conventional treatments. Another emerging area of research is the use of immunotherapy in targeting CSCs. Immunotherapies harness the body's immune system to recognize and eliminate CSCs. Strategies such as immune checkpoint inhibitors, chimeric antigen receptor (CAR) T-cell therapy, and cancer vaccines are being explored to stimulate the immune response against CSCs and improve treatment outcomes [120].

Treatment using CSCs holds promise for improving outcomes in cancer therapy. However, like other medical interventions, there are potential side effects and risks associated with this approach [121]. It is important to note that the risk of these side effects and complications is generally low but some of the reported side effects of CSC treatment include throat and mouth pain, vomiting, nausea, and the need for transfusions due to blood-related complications. Additionally, there is a risk of bleeding and infection, which are common concerns in any invasive medical procedure. Other potential risks specific to CSC treatment include hepatic hyperplasia [122, 123], and hepatic veno-occlusive disease [124]. However, precautionary measures should be taken to minimize the potential risks associated with the treatment. For instance, avoiding medications that suppress the immune system during the treatment period can positively impact the chances of successful cancerous tissue growth in the treated environment. To ensure the safety and effectiveness of CSC treatment, close monitoring, and careful management of potential side effects are necessary. Medical professionals and researchers continually work to improve the understanding of these risks and develop strategies to minimize them. By addressing and managing the side effects and risks associated with CSC treatment, the goal is to provide patients with safer and more effective therapeutic options for cancer management.

## 9. Role of CSCs for Immunization against Oncogenesis

CSCs play a crucial role in boosting the body's immune system to fight against cancer and are being extensively explored for their potential as vaccines. Recent research has revealed that introducing CSCs into the body can help combat growth-promoting proteins and stimulate the immune system to target various types of cancer [125]. In the human body, there are specialized cancer cells known as T-cancer cells that constantly survey the surface of cancerous tissues, examining them for any abnormality or potential threat to the body. Changes in the patterns of cancer cells, such as mutations or altered expression, can indicate the presence of viruses or bacteria. However, growing tumors have developed mechanisms to evade detection, making it challenging to identify and treat them at early stages [126]. This evasion weakens the body's immune response, allowing cancer to persist. Surgery is often considered the most effective method of removing tumors but it does not guarantee the complete eradication of all cancer cells. Residual cells can regenerate and contribute to disease recurrence. This emphasizes the need for complementary approaches that can strengthen the immune system and enhance its ability to recognize and eliminate cancer cells. CSC-based immunization strategies hold great promise in achieving this goal.

By harnessing the unique characteristics of CSCs, researchers aim to develop innovative therapies that target and activate the immune system against cancer. These efforts seek to overcome the challenges posed by tumor evasion mechanisms and improve the effectiveness of treatment by stimulating a robust immune response against cancerous cells. The use of CSC-based immunization approaches represents an exciting avenue in the ongoing battle against cancer, offering new opportunities for more effective and targeted treatments.

## 10. Clinical Trials of CSCs

It is well-understood that to eradicate diseased (malignant) cells, there must be a means to treat them with minimal adverse effects on normal cells. As a result, it is critical to strive to eradicate any malignant cancerous tissues that fuel the advancement of growth in the body in some way. The first medications have shown promising results in eliminating harmful malignant tissues. The issues being addressed include but are not limited to, antibody-mediated monotherapy that primarily targets CD47, as well as an antibody that targets ROR1. The Institute for Regenerative Medicine in California is conducting both of these trials (CIRM). It is carrying out the trial project by collaborating with other organizations to produce a unique type of stem cancerous tissue-based treatments that include therapy aimed at the elimination of malignant cancerous tissues [127]. Hundreds of studies have been listed on the clinical trials website of the US government (clinicaltrials.gov). Clinicians are attempting to increase the efficacy of treating children with aggressive neuroblastoma with high-dose chemotherapy and stem cell transplantation by combining chemotherapy, radiation, retinoids, immunotherapy, and other therapies [128]. Recent studies have indicated that doing two stem cell transplants (tandem transplants) in children with high-risk neuroblastoma is more successful than performing a single stem cell transplant [129–133]. They are testing if new chemotherapy medication combinations, such as busulfan and melphalan, are more successful than the ones typically used before a stem cell transplant. Other research is looking into whether utilizing stem cells supplied by someone else (allogeneic stem cell transplantation) rather than stem cells donated by the patient (autologous stem cell transplantation) may be more beneficial for children with difficult-to-treat cancers [134, 135]. Several studies are recruiting patients for adenocarcinoma treatment through CSCs (Table 3).

## 11. Current and Future Developments in CSCs

Current and future developments in CSC research are paving the way for significant advancements in our understanding of cancer biology and the development of innovative therapeutic approaches. One notable area of progress is the characterization of CSC markers, which involves identifying specific molecular signatures and genetic profiles associated with CSCs in various cancer types [134, 136]. This characterization enables researchers to isolate and study CSC populations, leading to a deeper understanding of their biology and behavior, and providing potential targets for therapeutic interventions. Another important aspect of CSC research is the exploration of CSC heterogeneity within tumors [135]. It is now recognized that CSC populations exhibit significant diversity, with distinct subpopulations displaying different characteristics and behaviors. Scientists are actively investigating the mechanisms underlying this heterogeneity and its implications for tumor growth, metastasis, and therapy resistance. Understanding CSC heterogeneity holds the key to developing targeted treatment strategies that address specific CSC subpopulations, thereby enhancing the effectiveness of therapies and reducing the likelihood of tumor recurrence.

Significant efforts are also being made to target CSC-specific signaling pathways [137, 138]. These pathways regulate key processes such as self-renewal, differentiation, and survival of CSCs. By targeting these pathways, researchers aim to disrupt CSC maintenance and inhibit tumor growth. Pathways such as notch, Wnt, and hedgehog have been identified as potential targets for therapeutic intervention [139]. Combination therapies that combine conventional treatments like chemotherapy and radiation with CSC-targeted therapies are being explored to enhance treatment efficacy. The goal is to simultaneously target both the bulk tumor cells and the CSC subpopulations, leading to better tumor regression, prevention of recurrence, and overcoming therapy resistance. Immunotherapies are also showing promise in the field of CSC research [140]. The immune system plays a crucial role in recognizing and eliminating cancer cells, including CSCs. Scientists are developing immunotherapeutic approaches, such as immune checkpoint inhibitors and adoptive cell therapies, to enhance the immune response against CSCs and improve patient outcomes.

Additionally, researchers are focusing on modulating the tumor microenvironment to disrupt CSC niches and their supportive interactions [141, 142]. The tumor microenvironment, including stromal cells, extracellular matrix components, and immune cells, plays a critical role in CSC regulation and tumor progression. Targeting the microenvironment holds potential as a therapeutic strategy to inhibit CSC growth and metastasis. Efforts are also underway to develop drugs specifically targeting CSCs, such as nanoparticle-mediated CSC destruction [119]. Specifically targeting drugs aim to selectively eliminate CSCs while sparing normal stem cells, reducing off-target effects, and improving treatment outcomes. Various approaches, such as small molecule inhibitors [143], and antibodies [144], apart from nanomedicine, are being explored for their potential to target CSC-specific pathways or markers. Advances in genomics and single-cell sequencing technologies are enabling the profiling of CSCs at a molecular level, leading to personalized medicine approaches. By understanding the genetic and epigenetic alterations specific to CSCs within an individual's tumor, tailored therapies can be designed to target the unique characteristics of their CSC populations, improving treatment outcomes and patient survival rates. The field of cancer stem cell research is rapidly evolving, with ongoing developments in CSC biology and therapeutic strategies. These advancements hold great promise for advancing our understanding of CSCs, improving cancer treatment outcomes, and ultimately offering more personalized and effective therapies for cancer patients. Continued research and collaboration in this area are vital to realizing the full potential of CSC-based approaches in cancer treatment.

## 12. Conclusion

Stem cell treatment has shown significant progress in the field of malignant tissue research, with continuous advancements being made through years of testing. Despite the existing challenges, each new experiment expands our understanding of the capabilities of stem cells in regenerative medicine and transplantology. The potential of cancer stem cell-based therapy to treat previously incurable diseases is remarkable. The ability to utilize cancerous tissues from patients, thanks to the development of pluripotency, has led to the establishment of tissue banks, which serve as a valuable resource for regenerative therapy in the fight against various diseases. The impact of cancer stem cell therapy on extending human life is unprecedented, offering promising prospects for the future. Treatments based on cancer stem cells represent one of the most exciting and promising areas of cancer research today.

## Figures and Tables

**Table 1:** Differences between cancer epithelial cells and CSCs.

Cancer epithelial cells	CSCs
Noninvasive with limited self-renewal potential and usually divide with a finite replicative capacity [24]	Invasive, migratory properties. CSCs exhibit self-renewal capabilities, allowing them to give rise to both identical CSCs and differentiated cancer cells, contributing to tumor perpetuation [25]

Typically more differentiated and closely resemble mature cell types [26]. They often form the bulk of the tumor	Less differentiated and exhibit properties akin to stem cells. They can differentiate into various cell types found within the tumor [25]

Cell polarity often responsible for initiating the tumor. They are derived from CSCs or non-CSC tumor cells [27].	CSCs have the unique ability to initiate tumor growth when transplanted into animal models, and they are considered the “seeds” of the tumor [28]

High expression of cell adhesion molecules [29]	Low (focal point) adhesion [30]

They usually display limited heterogeneity and represent the dominant, mature cell population within the tumor [31]	CSCs contribute to intratumoral heterogeneity by giving rise to both CSCs and differentiated cancer cells, resulting in a diverse cell population [32]

Nonmotile [33]	Highly mobile with stem cell-like behavior [25]

TGF*β* can lead to epithelial mesenchymal transition, promote metastasis and invasion [27]. Hence it can count as biomarker	CSCs express distinctive stem cell markers, including CD44, CD133, and specific transcription factors (e.g., OCT4, SOX2, NANOG), associated with pluripotency and self-renewal [34]

**Table 2:** Biomarkers of CSCs in human cancers [110–113].

Cancers	Markers	Function
Breast cancer	CD29^+^, CD49f^+^, CD90^+^, CD133^+^, Aldehyde Dehydrogenase^+^, ESA^+^/CD44^+^/CD24, CD44^+^/CD24^−^	Aldehyde Dehydrogenase: a chemical that helps the body's resilience CD44: a protein that is involved in cell movement and reproduction CD90: T-cell adherence and signaling pathways are both assisted by this protein CD133: a membrane protein that keeps lipids constituents inside the cellular membrane CD24: a biomarker that enables blood to flow through the tumor during metastasis CD49f: a component of the fibronectin group of protein complexes that has an involvement in cell attachment It plays a crucial role in cell attachment and communication

Prostate cancer	EpCAM^+^, CD117^+^, *α*2*β*1^+^, Aldehyde Dehydrogenase^+^, CD44^+^, EZH2^+^, CXCR4^+^, E-cadherin^+^, CD133^+^	*α*2*β*1: a cellular adhesive and identification receptor that has a role in cell attachment and identification E-cadherin is a molecule that promotes tumor movement and invasion CXCR4: this CXC-related protein binds the CD4 proteins to enable HIV to infiltrate organs EZH2: an important component of the nervous system as well as a part of the polycistronic group

Brain cancer	CD49f^+^, CD90^+^, CD44^+^, CD36^+^, EGFR^+^, A2B5^+^, L1 cell adhesion molecule^+^, CD133^+^,	CD36: the platelet's primary glycoprotein acts as just a binding protein EGFR: it interacts with fibroblast protein and promotes tumor proliferation A2B5: a glycoprotein biomarker that differentiates nerve subgroups in the nervous system L1 cell adhesion molecule: performs a vital role in the neurological cell motility and development

Stomach cancer	AldehydeDehydrogenase^+^, CD44^+^, CD44V8–10^+^, CD133^+^, CD24^+^, CD54^+^, CD90^+^, CD49f^+^ CD71^+^, EpCAM^+^	CD44V8–10: a CD44 variant related to a subpopulation of tumorigenesis CD54: belongs to a family of adhesive proteins and produces cancer cells

Colorectal cancer	CD200^+^, EpCAM^+^, CD133^+^, CD166^+^, CD206^+^, CD44^+^, CD49f^+^, AldehydeDehydrogenase^+^	CD200: tt has a role in immunoregulation CD166: it interacts with a 6th edition T-cell differentiation marker and helps in cell proliferation and migration CD206: cellular membranes, clearance, and immune homeostasis all are assisted by the mannuronic receptors EpCAM: this is a cell–cell nutrient cellular adhesion molecule that is also present over most epithelia and in intestinal malignancies

Liver cancer	CD24^+^, CD133^+^, CD13^+^, CD44^+^, CD206^+^, OV-6^+^, CD90^+^, EpCAM^+^	CD13: glycoprotein involved in protein metabolism OV-6: cell surface antigen and used as a biomarker
AML cancer	CD34^+^, CD38^−^, CD90^+^, CD71^+^, CD19^+^, CD20^+^, CD44^+^, CD10^+^, CD45RA^+^, CD123^+^	CD34: tt helps the adhesion of cell lines to external or stroma polymorphonuclear leukocytes CD38: predictive factor for individuals with persistent lymphoblastic leukemia, an internal Ca2+ recruitment pathway CD71: for neuron development, serum ferritin receptors are needed for cellular iron uptake CD19: phagocytic cell maturation is governed by a group of signaling. pathway component, including CD19 CD20: the protein is essential for B cell development and differentiation to lymphoid cells CD10: it is a neutral endopeptidase which inactivates several peptides CD45RA: the CD45 antigen is a receptor protein with tyrosine phosphatase activity, also known as the Ly-5 or leukocyte common antigen CD123: a component of a homodimer cytokines sensor that is particular for cytokines

Melanoma cancer	CD20^+^, CD271^+^, AldehydeDehydrogenase^+^, CD133^+^	CD271 marker to identify mesenchymal stem cells. It mediates the stemness of melanoma cells and serves as a regulator of metastasis

Bladder cancer	CD44v6^+^, CD44^+^, ALDEHYDE DEHYDROGENASE^+^	CD44v6 is involved in cell cycle progression and cell attachment

Ovarian cancer	CD24^+^, AldehydeDehydrogenase^+^, CD44^+^/CD117^+^, EpCAM^+^, CD133^+^	CD117 expression has a pathogenic role in many malignancies, including ovarian carcinoma

Pancreas cancer	Aldehyde Dehydrogenase^+^, CD133^+^, CD44^+^/CD24^+^/EpCAM^+^, ABCG2^+^, CXCR4^+^,	ABCG2: the ABC carrier subfamily is a set of biological membranes that serve as a factor in the antibiotic resistance of CSCs

Head and neck squamous cell carcinomas cancer	Aldehyde Dehydrogenase^+^, CD44^+^, CD166^+^	–
Gallbladder cancer	CD44^+^/CD133^+^	–

Renal cell carcinoma cancer	CD133^+^, AldehydeDehydrogenase^+^, CXCR4^+^, CD44^+^, CD105^+^	CD105: it is involved in the regulation of angiogenesis, the process of forming new blood vessels. Prognostic marker in renal cancer

Lung cancer	CD166^+^, CD90^+^, CD87^+^, AldehydeDehydrogenase^+^, CD44^+^, CD133^+^	CD87: connected to cell membrane plasmin stimulation and local degradation of extracellular environment. It is allied with many physiologic and pathologic events

Malignant mesothelioma Cancer	CD9^+^, CD24^+^, CD26^+^	CD9: glycosylated protein is involved in a range of cellular processes, including such division, attachment, and signal transduction, as well as cancerous cells motion and metastasis CD26: involved in glucose metabolism and immune system modulationHighly expressed in malignant mesothelioma compared to the benign tissues

Oral squamous cell carcinoma cancer	CD44^+^/CD24,^−^ ITGA7^+^	ITGA7: an adhesion molecule related to the cell relocation, morphogenesis, distinction, metastatic spread, delineation, and relocation during early embryogenesis

Cutaneous squamous cell carcinoma cancer	CD44^+^, CD133^+^	–
Esophageal cancer	ITGA7^+^, CD44^+^, AldehydeDehydrogenase^+^, CD133^+^, CD90^+^	–
Multiple myeloma cancer	CD138−, CD19^+^, CD27^+^,	CD138: it is involved in cell–cell adhesion, cell migration, differentiation of plasma cells, and cell signaling processes CD27: CD27 acts as a co-stimulatory molecule for T cells, enhancing their activation and proliferation

Cervix cancer	ABCG2^+^, CD133^+^, CD49f^+^, AldehydeDehydrogenase^+^	–

Nasopharyngeal cancer	CD44^+^, CD133^+^, AldehydeDehydrogenase^+^, CD24^+^	–

Laryngeal cancer	Aldehyde Dehydrogenase^+^, CD44^+^, CD133^+^	–

**Table 3:** Ongoing clinical trials recruiting adenocarcinoma patients for CSC-based trials (source: clinicaltrials.gov; retrieved 20 May 2023).

Serial no.	Conditions	Interventions	Trial number
1	Gastric and cardia adenocarcinomas	Procedure: biopsy	NCT02491840

2	Pancreas cancer	Drug: bethanechol	NCT03572283

3	Resectable pancreatic adenocarcinoma	Drug: HIPEC-gemcitabine	NCT03251365

4	Adenocarcinoma of lung	Genetic: MSCTRAIL|Drug: placebo	NCT03298763

5	Pancreatic adenocarcinoma metastatic|BRCA1 mutation|BRCA2 mutation|Pancreatic acinar cell carcinoma|Pancreatic ductal adenocarcinoma|Pancreatic cancer|Metastatic pancreatic cancer|Metastatic pancreatic ductal adenocarcinoma|Breast cancer metastatic|Breast cancer stage IV|Pancreatic cancer stage IV|HER2-negative breast cancer|HER2 negative breast carcinoma|Adenocarcinoma of the breast|PALB2 gene mutation	Drug: melphalan|Drug: BCNU|Drug: vitamin B12B|Drug: vitamin C|Drug: ethanol|Device: autologous hematopoietic stem cells	NCT04150042

6	Fallopian tube clear cell adenocarcinoma|Fallopian tube endometrioid adenocarcinoma|Fallopian tube mucinous adenocarcinoma|Fallopian tube serous adenocarcinoma|Fallopian tube transitional cell carcinoma|Fallopian tube undifferentiated carcinoma|Malignant ovarian brenner tumor|Ovarian clear cell adenocarcinoma|Ovarian endometrioid adenocarcinoma|Ovarian mucinous adenocarcinoma|Ovarian seromucinous carcinoma|Ovarian serous adenocarcinoma|Ovarian transitional cell carcinoma|Ovarian undifferentiated carcinoma|Primary peritoneal serous adenocarcinoma|Recurrent fallopian tube carcinoma|Recurrent ovarian carcinoma|Recurrent primary peritoneal carcinoma	Other: laboratory biomarker analysis|Procedure: mesenchymal stem cell transplantation|Biological: oncolytic measles virus encoding thyroidal sodium iodide symporter	NCT02068794

7	Pancreatic ductal adenocarcinoma|Metastatic pancreatic cancer|Circulating tumor cell	Other: detection of circulating tumor cells expressing Axl: CTC-AXL(+)	NCT05346536

8	Pancreatic cancer|Metastatic pancreatic cancer|Metastatic pancreatic adenocarcinoma	Drug: zolbetuximab|Drug: nab-paclitaxel|Drug: gemcitabine	NCT03816163

9	Colorectal cancer metastases and hepatocellular carcinomas	Procedure: tumor /metastases removal	NCT05384184

10	Rectum adenocarcinoma	Other: biopsy	NCT02849158

11	Central nervous system nongerminomatous germ cell tumor|Choriocarcinoma|Embryonal carcinoma|Immature teratoma|Malignant teratoma|Mixed germ cell tumor|Pineal region germ cell tumor|Pineal region immature teratoma|Pineal region yolk sac tumor|Suprasellar germ cell tumor	Drug: carboplatin|Drug: etoposide|Biological: filgrastim|Drug: ifosfamide|Drug: mesna|Biological: pegfilgrastim|Procedure: peripheral blood stem cell transplantation|Other: questionnaire administration|Radiation: radiation therapy|Procedure: second-look surgery|Drug: thiotepa	NCT04684368

12	Hepatocellular carcinoma	Drug: peginterferon *α*-2b (pegabin) with entecavir or tenofovir fumarate or propofol tenofovir fumarate | Procedure: radical surgery	NCT05466565

## References

[1] Ghosh S., Juin S. K., Majumdar S. B., Majumdar S. (2021). Crucial role of glucosylceramide synthase in the regulation of stem cell-like cancer cells in B16F10 murine melanoma. *Molecular Carcinogenesis*.

[2] Atashzar M. R., Baharlou R., Karami J. (2020). Cancer stem cells: a review from origin to therapeutic implications. *Journal of Cellular Physiology*.

[3] Clarke M. F. (2019). Clinical and therapeutic implications of cancer stem cells. *New England Journal of Medicine*.

[4] Wan Kamarul Zaman W. S., Nurul A. A., Nordin F. (2021). Stem cells and cancer stem cells: the Jekyll and Hyde scenario and their implications in stem cell therapy. *Biomedicines*.

[5] Pineda J. R., Badiola I., Ibarretxe G. (2021). Stem and cancer stem cell identities, cellular markers, niche environment and response to treatments to unravel new therapeutic targets. *Biology*.

[6] Echegaray C. V., Guberman A. S. (2022). Nanog in iPS cells and during reprogramming. *Molecular Players in iPSC Technology*.

[7] Wei S., Li J., Tang M. (2022). STAT3 and p63 in the regulation of cancer stemness. *Frontiers in Genetics*.

[8] Holland J. D., Klaus A., Garratt A. N., Birchmeier W. (2013). Wnt signaling in stem and cancer stem cells. *Current Opinion in Cell Biology*.

[9] Takebe N., Harris P. J., Warren R. Q., Ivy S. P. (2011). Targeting cancer stem cells by inhibiting Wnt, Notch, and Hedgehog pathways. *Nature Reviews Clinical Oncology*.

[10] Robinson N. J. (2020). *Uncovering the Dynamic Regulation of Telomeres in Cancer by SLX4IP*.

[11] Yadav R. P., Baranwal S. (2024). Kindlin-2 regulates colonic cancer stem-like cells survival and self-renewal via Wnt/*β*-catenin mediated pathway. *Cellular Signalling*.

[12] Fidoamore A., Cristiano L., Laezza C. (2017). Energy metabolism in glioblastoma stem cells: PPAR*α* a metabolic adaptor to intratumoral microenvironment. *Oncotarget*.

[13] Chandler J. M., Lagasse E. (2010). Cancerous stem cells: deviant stem cells with cancer-causing misbehavior. *Stem Cell Research & Therapy*.

[14] Zheng X., Yu C., Xu M. (2021). Linking tumor microenvironment to plasticity of cancer stem cells: mechanisms and application in cancer therapy. *Frontiers in Oncology*.

[15] Zarrintaj P., Mostafapoor F., Milan P. B., Saeb M. R. (2019). Theranostic platforms proposed for cancerous stem cells: a review. *Current Stem Cell Research & Therapy*.

[16] Shipitsin M., Polyak K. (2008). The cancer stem cell hypothesis: in search of definitions, markers, and relevance. *Laboratory Investigation*.

[17] Chu D.-T., Nguyen T. T., Tien N. L. B. (2020). Recent progress of stem cell therapy in cancer treatment: molecular mechanisms and potential applications. *Cells*.

[18] Barakat B., Franke K., Schakaki S., Hijazi S., Hasselhof V., Vögeli T.-A. (2020). Stem cell applications in regenerative medicine for stress urinary incontinence: a review of effectiveness based on clinical trials. *Arab Journal of Urology*.

[19] Melick G., Hayman N., Landsman A. S. (2018). Mesenchymal stem cell applications for joints in the foot and ankle. *Clinics in Podiatric Medicine and Surgery*.

[20] Liu G., David B. T., Trawczynski M., Fessler R. G. (2020). Advances in pluripotent stem cells: history, mechanisms, technologies, and applications. *Stem Cell Reviews and Reports*.

[21] Thompson E. J. Till, McCulloch E. (2020). *The Team that Discovered Stem Cells*.

[22] Sornberger J. (2011). *Dreams and Due Diligence: Till and McCulloch’s Stem Cell Discovery and Legacy*.

[23] Rossi F., Noren H., Jove R., Beljanski V., Grinnemo K.-H. (2020). Differences and similarities between cancer and somatic stem cells: therapeutic implications. *Stem Cell Research & Therapy*.

[24] Kyo S., Maida Y., Inoue M. (2011). Stem cells in endometrium and endometrial cancer: accumulating evidence and unresolved questions. *Cancer Letters*.

[25] Ayob A. Z., Ramasamy T. S. (2018). Cancer stem cells as key drivers of tumour progression. *Journal of Biomedical Science*.

[26] van Leenders G. J. L. H., Schalken J. A. (2003). Epithelial cell differentiation in the human prostate epithelium: implications for the pathogenesis and therapy of prostate cancer. *Critical Reviews in Oncology/Hematology*.

[27] Royer C., Lu X. (2011). Epithelial cell polarity: a major gatekeeper against cancer?. *Cell Death & Differentiation*.

[28] Visvader J. E., Lindeman G. J. (2008). Cancer stem cells in solid tumours: accumulating evidence and unresolved questions. *Nature Reviews Cancer*.

[29] Simon M., Stefan N., Plückthun A., Zangemeister-Wittke U. (2013). Epithelial cell adhesion molecule-targeted drug delivery for cancer therapy. *Expert Opinion on Drug Delivery*.

[30] Tan K. K. B., Giam C. S. Y., Leow M. Y., Chan C. W., Yim E. K. F. (2015). Differential cell adhesion of breast cancer stem cells on biomaterial substrate with nanotopographical cues. *Journal of Functional Biomaterials*.

[31] Marjanovic N. D., Weinberg R. A., Chaffer C. L. (2013). Cell plasticity and heterogeneity in cancer. *Clinical Chemistry*.

[32] Clarke M., Frampton J. (2020). *Stem Cells: Biology and Application*.

[33] Prieto-García E., Díaz-García C. V., García-Ruiz I., Agulló-Ortuño M. T. (2017). Epithelial-to-mesenchymal transition in tumor progression. *Medical Oncology*.

[34] Wang M., Chiou S., Wu C. (2013). Targeting cancer stem cells: emerging role of Nanog transcription factor. *OncoTargets and Therapy*.

[35] Ho T.-C., LaMere M., Stevens B. M. (2016). Evolution of acute myelogenous leukemia stem cell properties after treatment and progression. *Blood*.

[36] Katoh M. (2017). Canonical and non-canonical WNT signaling in cancer stem cells and their niches: cellular heterogeneity, omics reprogramming, targeted therapy and tumor plasticity (review). *International Journal of Oncology*.

[37] Al-Hajj M., Wicha M. S., Benito-Hernandez A., Morrison S. J., Clarke M. F. (2003). Prospective identification of tumorigenic breast cancer cells. *Proceedings of the National Academy of Sciences*.

[38] Hemmati H. D., Nakano I., Lazareff J. A. (2003). Cancerous stem cells can arise from pediatric brain tumors. *Proceedings of the National Academy of Sciences*.

[39] Ricci-Vitiani L., Lombardi D. G., Pilozzi E. (2007). Identification and expansion of human colon-cancer-initiating cells. *Nature*.

[40] Prince M. E., Sivanandan R., Kaczorowski A. (2007). Identification of a subpopulation of cells with cancer stem cell properties in head and neck squamous cell carcinoma. *Proceedings of the National Academy of Sciences*.

[41] Kim C. F. B., Jackson E. L., Woolfenden A. E. (2005). Identification of bronchioalveolar stem cells in normal lung and lung cancer. *Cell*.

[42] Li C., Heidt D. G., Dalerba P. (2007). Identification of pancreatic cancer stem cells. *Cancer Research*.

[43] Monzani E., Facchetti F., Galmozzi E. (2007). Melanoma contains CD133 and ABCG2 positive cells with enhanced tumourigenic potential. *European Journal of Cancer*.

[44] Jones R. J., Matsui W. H., Smith B. D. (2004). Cancer stem cells: are we missing the target?. *JNCI Journal of the National Cancer Institute*.

[45] Bhatia R., Holtz M., Niu N. (2003). Persistence of malignant hematopoietic progenitors in chronic myelogenous leukemia patients in complete cytogenetic remission following imatinib mesylate treatment. *Blood*.

[46] Graham S. M., Jørgensen H. G., Allan E. (2002). Primitive, quiescent, philadelphia-positive stem cells from patients with chronic myeloid leukemia are insensitive to STI571 in vitro. *Blood*.

[47] Collins A. T., Berry P. A., Hyde C., Stower M. J., Maitland N. J. (2005). Prospective identification of tumorigenic prostate cancer stem cells. *Cancer Research*.

[48] Nimmakayala R. K., Batra S. K., Ponnusamy M. P. (2019). Unraveling the journey of cancer stem cells from origin to metastasis. *Biochimica et Biophysica Acta(BBA)-Reviews on Cancer*.

[49] Cox C. V., Evely R. S., Oakhill A., Pamphilon D. H., Goulden N. J., Blair A. (2004). Characterization of acute lymphoblastic leukemia progenitor cells. *Blood*.

[50] Duy C., Li M., Teater M. (2021). Chemotherapy induces senescence-like resilient cells capable of initiating AML recurrence senescence-like cells contribute to relapse of AML. *Cancer Discovery*.

[51] Park S.-Y., Nam J.-S. (2020). The force awakens: metastatic dormant cancer cells. *Experimental & Molecular Medicine*.

[52] Sabisz M., Skladanowski A. (2009). Cancer stem cells and escape from drug-induced premature senescence in human lung tumor cells: implications for drug resistance and in vitro drug screening models. *Cell Cycle*.

[53] Zhang D. Y., Monteiro M. J., Liu J.-P., Gu W.-Y. (2021). Mechanisms of cancer stem cell senescence: current understanding and future perspectives. *Clinical and Experimental Pharmacology and Physiology*.

[54] Kim H. J., Park J.-S. (2017). Usage of human mesenchymal stem cells in cell-based therapy: advantages and disadvantages. *Development & Reproduction*.

[55] Li G., Chen Z., Hu Y.-D. (2009). Autocrine factors sustain glioblastoma stem cell self-renewal. *Oncology Reports*.

[56] Ma B., Lei X., Guan Y. (2011). Maintenance of retinal cancer stem cell-like properties through long-term serum-free culture from human retinoblastoma. *Oncology Reports*.

[57] Wang X., Huang S., Chen J.-L. (2017). Understanding of leukemic stem cells and their clinical implications. *Molecular Cancer*.

[58] Sallman D. A., Asch A. S., Al Malki M. M. (2019). The first-in-class anti-CD47 antibody magrolimab (5F9) in combination with azacitidine is effective in MDS and AML patients: ongoing phase 1b results. *Blood*.

[59] Fang D. D., Kim Y. J., Lee C. N. (2010). Expansion of CD133+ colon cancer cultures retaining stem cell properties to enable cancer stem cell target discovery. *British Journal of Cancer*.

[60] Sikic B. I., Lakhani N., Patnaik A. (2019). First-in-human, first-in-class phase I trial of the anti-CD47 antibody Hu5F9-G4 in patients with advanced cancers. *Journal of Clinical Oncology*.

[61] Thomas D., Majeti R. (2017). Biology and relevance of human acute myeloid leukemia stem cells. *Blood*.

[62] Najafi M., Farhood B., Mortezaee K. (2019). Cancer stem cells (CSCs) in cancer progression and therapy. *Journal of Cellular Physiology*.

[63] Celià-Terrassa T., Liu D. D., Choudhury A. (2017). Normal and cancerous mammary stem cells evade interferon-induced constraint through the miR-199a–LCOR axis. *Nature Cell Biology*.

[64] Wayne E. C., Chandrasekaran S., Mitchell M. J. (2016). TRAIL-coated leukocytes that prevent the bloodborne metastasis of prostate cancer. *Journal of Controlled Release*.

[65] Li P., Xing W., Xu J. (2019). microRNA-301b-3p downregulation underlies a novel inhibitory role of long non-coding RNA MBNL1-AS1 in non-small cell lung cancer. *Stem Cell Research & Therapy*.

[66] Cheng J.-T., Wang L., Wang H. (2019). Insights into biological role of LncRNAs in epithelial–mesenchymal transition. *Cells*.

[67] Yeung T. M., Gandhi S. C., Bodmer W. F. (2011). Hypoxia and lineage specification of cell line-derived colorectal cancer stem cells. *Proceedings of the National Academy of Sciences*.

[68] Ding K., Banerjee A., Tan S. (2014). Artemin, a member of the glial cell line-derived neurotrophic factor family of ligands, is HER2-regulated and mediates acquired trastuzumab resistance by promoting cancer stem cell-like behavior in mammary carcinoma cells. *Journal of Biological Chemistry*.

[69] Poggi A., Villa F., Fernadez J. L. C., Costa D., Zocchi M. R., Benelli R. (2021). Three-dimensional culture models to study innate anti-tumor immune response: advantages and disadvantages. *Cancers*.

[70] Stribbling S. M., Ryan A. J. (2022). The cell-line-derived subcutaneous tumor model in preclinical cancer research. *Nature Protocols*.

[71] Moro M., Bertolini G., Tortoreto M., Pastorino U., Sozzi G., Roz L. (2012). Patient-derived xenografts of non small cell lung cancer: resurgence of an old model for investigation of modern concepts of tailored therapy and cancer stem cells. *Journal of Biomedicine and Biotechnology*.

[72] Keysar S. B., Eagles J. R., Miller B. (2018). Salivary gland cancer patient-derived xenografts enable characterization of cancer stem cells and new gene events associated with tumor progressionsalivary cancer stem cells increase with disease progression. *Clinical Cancer Research*.

[73] Pizon M., Schott D., Pachmann U. (2022). Chick chorioallantoic membrane (CAM) assays as a model of patient-derived xenografts from circulating cancer stem cells (cCSCs) in breast cancer patients. *Cancers*.

[74] Chao H.-M., Chern E. (2018). Patient-derived induced pluripotent stem cells for models of cancer and cancer stem cell research. *Journal of the Formosan Medical Association*.

[75] Williams J. A. (2018). Using PDX for preclinical cancer drug discovery: the evolving field. *Journal of Clinical Medicine*.

[76] Wilson H. V. (1907). A new method by which sponges may be artificially reared. *Science*.

[77] Sachs N., de Ligt J., Kopper O. (2018). A living biobank of breast cancer organoids captures disease heterogeneity. *Cell*.

[78] Li Z., Qian Y., Li W. (2020). Human lung adenocarcinoma-derived organoid models for drug screening. *iScience*.

[79] Crespo M., Vilar E., Tsai S.-Y. (2017). Colonic organoids derived from human induced pluripotent stem cells for modeling colorectal cancer and drug testing. *Nature Medicine*.

[80] Driehuis E., van Hoeck A., Moore K. (2019). Pancreatic cancer organoids recapitulate disease and allow personalized drug screening. *Proceedings of the National Academy of Sciences*.

[81] Wuputra K., Ku C.-C., Kato K., Wu D.-C., Saito S., Yokoyama K. K. (2021). Translational models of 3-D organoids and cancer stem cells in gastric cancer research. *Stem Cell Research & Therapy*.

[82] Qu J., Kalyani F. S., Liu L., Cheng T., Chen L. (2021). Tumor organoids: synergistic applications, current challenges, and future prospects in cancer therapy. *Cancer Communications*.

[83] Chen S.-F., Chang Y.-C., Nieh S. (2012). Nonadhesive culture system as a model of rapid sphere formation with cancer stem cell properties. *PLOS ONE*.

[84] Zhang C., Yang Z., Dong D.-L. (2020). 3D culture technologies of cancer stem cells: promising ex vivo tumor models. *Journal of Tissue Engineering*.

[85] Bielecka Z. F., Maliszewska-Olejniczak K., Safir I. J., Szczylik C., Czarnecka A. M. (2017). Three-dimensional cell culture model utilization in cancer stem cell research. *Biological Reviews*.

[86] Vias M., Gavarró L. M., Sauer C. M. (2023). High-grade serous ovarian carcinoma organoids as models of chromosomal instability. *Elife*.

[87] Papaccio F., García-Mico B., Gimeno-Valiente F. (2023). “Proteotranscriptomic analysis of advanced colorectal cancer patient derived organoids for drug sensitivity prediction”. *Journal of Experimental & Clinical Cancer Research*.

[88] LeSavage B. L., Suhar R. A., Broguiere N., Lutolf M. P., Heilshorn S. C. (2022). Next-generation cancer organoids. *Nature Materials*.

[89] Rossi G., Manfrin A., Lutolf M. P. (2018). Progress and potential in organoid research. *Nature Reviews Genetics*.

[90] Bose S., Clevers H., Shen X. (2021). Promises and challenges of organoid-guided precision medicine. *Med*.

[91] Shibata M., Shen M. M. (2015). Stem cells in genetically-engineered mouse models of prostate cancer. *Endocrine-Related Cancer*.

[92] Singh M., Murriel C. L., Johnson L. (2012). Genetically engineered mouse models: closing the gap between preclinical data and trial outcomes. *Cancer Research*.

[93] Ishiguro T., Ohata H., Sato A., Yamawaki K., Enomoto T., Okamoto K. (2017). Tumor-derived spheroids: relevance to cancer stem cells and clinical applications. *Cancer Science*.

[94] Guerrero-Cázares H., Chaichana K. L., Quiñones-Hinojosa A. (2009). Neurosphere culture and human organotypic model to evaluate brain tumor stem cells. *Cancer Stem Cells*.

[95] Costea D. E., Tsinkalovsky O., Vintermyr O. K., Johannessen A. C., Mackenzie I. C. (2006). Cancer stem cellsnew and potentially important targets for the therapy of oral squamous cell carcinoma. *Oral Diseases*.

[96] Kim M., Mun H., Sung C. O. (2019). Patient-derived lung cancer organoids as in vitro cancer models for therapeutic screening. *Nature Communications*.

[97] Azizipour N., Avazpour R., Rosenzweig D. H., Sawan M., Ajji A. (2020). Evolution of biochip technology: a review from lab-on-a-chip to organ-on-a-chip. *Micromachines*.

[98] Dogan E., Kisim A., Bati-Ayaz G., Kubicek G. J., Pesen-Okvur D., Miri A. K. (2021). Cancer stem cells in tumor modeling: challenges and future directions. *Advanced NanoBiomed Research*.

[99] Samadian H., Jafari S., Sepand M. R. (2021). 3D bioprinting technology to mimic the tumor microenvironment: tumor-on-a-chip concept. *Materials Today Advances*.

[100] Huh D., Hamilton G. A., Ingber D. E. (2011). From 3D cell culture to organs-on-chips. *Trends in Cell Biology*.

[101] Probst C., Schneider S., Loskill P. (2018). High-throughput organ-on-a-chip systems: current status and remaining challenges. *Current Opinion in Biomedical Engineering*.

[102] Lee J., Kotliarova S., Kotliarov Y. (2006). Tumor stem cells derived from glioblastomas cultured in bFGF and EGF more closely mirror the phenotype and genotype of primary tumors than do serum-cultured cell lines. *Cancer Cell*.

[103] Wang R., Lv Q., Meng W. (2014). Comparison of mammosphere formation from breast cancer cell lines and primary breast tumors. *Journal of Thoracic Disease*.

[104] AlMusawi S., Ahmed M., Nateri A. S. (2021). Understanding cell–cell communication and signaling in the colorectal cancer microenvironment. *Clinical and Translational Medicine*.

[105] Di Liello R., Ciaramella V., Barra G. (2019). Ex vivo lung cancer spheroids resemble treatment response of a patient with NSCLC to chemotherapy and immunotherapy: case report and translational study. *ESMO Open*.

[106] Hickman J. A., Graeser R., de Hoogt R. (2014). Three-dimensional models of cancer for pharmacology and cancer cell biology: capturing tumor complexity in vitro/ex vivo. *Biotechnology Journal*.

[107] Han S. J., Kwon S., Kim K. S. (2021). Challenges of applying multicellular tumor spheroids in preclinical phase. *Cancer Cell International*.

[108] Zhou B.-B. S., Zhang H., Damelin M., Geles K. G., Grindley J. C., Dirks P. B. (2009). Tumour-initiating cells: challenges and opportunities for anticancer drug discovery. *Nature Reviews Drug Discovery*.

[109] Galardi S., Savino M., Scagnoli F. (2016). Resetting cancer stem cell regulatory nodes upon MYC inhibition. *EMBO Reports*.

[110] Ochiya T., Takahashi R.-u. (2018). MicroRNAs: novel biomarkers and therapeutic targets for human cancers.

[111] Barh D., Carpi A., Verma M., Gunduz M. (2014). *Cancer B iomarke rs: Minimal and Noninvasive Early Diagnosis and Prognosis*.

[112] Bogler O., Sawaya R. (2008). Foreword. *Current Problems in Cancer*.

[113] Stobezki R. (2010). *Study of Biomarkers of Invasion and Cancer Stem Cells in Human Breast Cancer*.

[114] El Hout M., Dos Santos L., Hamaï A., Mehrpour M. (2018). A promising new approach to cancer therapy: Targeting iron metabolism in cancer stem cells. *Seminars in Cancer Biology*.

[115] Croker A. K., Allan A. L. (2008). Cancer stem cells: implications for the progression and treatment of metastatic disease. *Journal of Cellular and Molecular Medicine*.

[116] Koltai H., Shalev N. (2022). Anti-cancer activity of *cannabis sativa* phytocannabinoids: molecular mechanisms and potential in the fight against ovarian cancer and stem cells. *Cancers*.

[117] Bian J., Fu J., Wang X. (2022). Characterization of immunogenicity of malignant cells with stemness in intrahepatic cholangiocarcinoma by single-cell RNA sequencing. *Stem Cells International*.

[118] Bagheri-Mohammadi S. (2022). Adult neurogenesis and the molecular signalling pathways in brain: the role of stem cells in adult hippocampal neurogenesis. *International Journal of Neuroscience*.

[119] Shen S., Xia J.-X., Wang J. (2016). Nanomedicine-mediated cancer stem cell therapy.. *Biomaterials*.

[120] Masoumi J., Jafarzadeh A., Abdolalizadeh J. (2021). Cancer stem cell-targeted chimeric antigen receptor (CAR)-T cell therapy: challenges and prospects. *Acta Pharmaceutica Sinica B*.

[121] Zhang C.-L., Huang T., Wu B.-L., He W.-X., Liu D. (2017). Stem cells in cancer therapy: opportunities and challenges. *Oncotarget*.

[122] Benderitter M., Caviggioli F., Chapel A. (2014). Stem cell therapies for the treatment of radiation-induced normal tissue side effects. *Antioxidants & Redox Signaling*.

[123] Masetti R., Biagi C., Kleinschmidt K. (2011). Focal nodular hyperplasia of the liver after intensive treatment for pediatric cancer: is hematopoietic stem cell transplantation a risk factor?. *European Journal of Pediatrics*.

[124] Echart C., Repice C., Ferro L. (2007). Reduced fibrinolysis in the hepatic venous occlusive disease: effect of defibrotide on plasmin activity. *Blood*.

[125] Nazio F., Bordi M., Cianfanelli V., Locatelli F., Cecconi F. (2019). Autophagy and cancer stem cells: molecular mechanisms and therapeutic applications. *Cell Death & Differentiation*.

[126] Annett S., Robson T. (2018). Targeting cancer stem cells in the clinic: current status and perspectives. *Pharmacology & Therapeutics*.

[127] Tran C., Damaser M. S. (2015). Stem cells as drug delivery methods: application of stem cell secretome for regeneration. *Advanced Drug Delivery Reviews*.

[128] Seo E. S., Shin M., Lim H. (2022). Clinical implication of residual MIBG-positive disease in the follow-up of high-risk neuroblastoma treated with tandem high-dose chemotherapy and autologous stem cell transplantation. *Pediatric Blood Cancer*.

[129] Park J. R., Kreissman S. G., London W. B. (2016). A phase III randomized clinical trial (RCT) of tandem myeloablative autologous stem cell transplant (ASCT) using peripheral blood stem cell (PBSC) as consolidation therapy for high-risk neuroblastoma (HR-NB): a Children’s Oncology Group (COG) study. *Journal of Clinical Oncology*.

[130] Hobbie W. L., Moshang T., Carlson C. A. (2008). Late effects in survivors of tandem peripheral blood stem cell transplant for high-risk neuroblastoma. *Pediatric Blood Cancer*.

[131] Seif A. E., Naranjo A., Baker D. L. (2013). A pilot study of tandem high-dose chemotherapy with stem cell rescue as consolidation for high-risk neuroblastoma: children’s Oncology Group study ANBL00P1. *Bone Marrow Transplantation*.

[132] Pasqualini C., Dufour C., Goma G., Raquin M.-A., Lapierre V., Valteau-Couanet D. (2016). Tandem high-dose chemotherapy with thiotepa and busulfan–melphalan and autologous stem cell transplantation in very high-risk neuroblastoma patients. *Bone Marrow Transplantation*.

[133] Suh J. K., Koh K.-Nam, Min S. Y. (2020). Feasibility and effectiveness of treatment strategy of tandem high-dose chemotherapy and autologous stem cell transplantation in combination with ^131^ I-MIBG therapy for high-risk neuroblastoma. *Pediatric Transplantation*.

[134] Shrivastava S., Steele R., Sowadski M., Crawford S. E., Varvares M., Ray R. B. (2015). Identification of molecular signature of head and neck cancer stem-like cells. *Scientific Reports*.

[135] Zheng H., Pomyen Y., Hernandez M. O. (2018). Single-cell analysis reveals cancer stem cell heterogeneity in hepatocellular carcinoma. *Hepatology*.

[136] Zakaria N., Yusoff N. M., Zakaria Z. (2015). Human non-small cell lung cancer expresses putative cancer stem cell markers and exhibits the transcriptomic profile of multipotent cells. *BMC Cancer*.

[137] Ranji P., Salmani Kesejini T., Saeedikhoo S., Alizadeh A. M. (2016). Targeting cancer stem cell-specific markers and/or associated signaling pathways for overcoming cancer drug resistance. *Tumor Biology*.

[138] Leon G., MacDonagh L., Finn S. P., Cuffe S., Barr M. P. (2016). Cancer stem cells in drug resistant lung cancer: targeting cell surface markers and signaling pathways. *Pharmacology & Therapeutics*.

[139] Agliano A., Calvo A., Box C. (2017). The challenge of targeting cancer stem cells to halt metastasis. *Seminars in Cancer Biology*.

[140] Daher M., Rezvani K. (2018). Next generation natural killer cells for cancer immunotherapy: the promise of genetic engineering. *Current Opinion in Immunology*.

[141] Park T. S., Donnenberg V. S., Donnenberg A. D., Zambidis E. T., Zimmerlin L. (2014). Dynamic interactions between cancer stem cells and their stromal partners. *Current Pathobiology Reports*.

[142] Emami Nejad A., Najafgholian S., Rostami A. (2021). The role of hypoxia in the tumor microenvironment and development of cancer stem cell: a novel approach to developing treatment. *Cancer Cell International*.

[143] De K., Grubb T. M., Zalenski A. A. (2019). Hyperphosphorylation of CDH1 in Glioblastoma cancer stem cells attenuates APC/CCDH1 activity and pharmacologic inhibition of APC/CCDH1/CDC20 compromises viability dysregulation and targeting of the APC/C in GBM CSCs. *Molecular Cancer Research*.

[144] Boesch M., Spizzo G., Seeber A. (2018). Concise review: aggressive colorectal cancer: role of epithelial cell adhesion molecule in cancer stem cells and epithelial-to-mesenchymal transition. *Stem Cells Translational Medicine*.

